# Increase of invasive meningococcal serogroup W disease in Europe, 2013 to 2017

**DOI:** 10.2807/1560-7917.ES.2019.24.14.1800245

**Published:** 2019-04-04

**Authors:** Manuel Krone, Steve Gray, Raquel Abad, Anna Skoczyńska, Paola Stefanelli, Arie van der Ende, Georgina Tzanakaki, Paula Mölling, Maria João Simões, Pavla Křížová, Stéphane Emonet, Dominique A. Caugant, Maija Toropainen, Julio Vazquez, Izabela Waśko, Mirjam J. Knol, Susanne Jacobsson, Célia Rodrigues Bettencourt, Martin Musilek, Rita Born, Ulrich Vogel, Ray Borrow

**Affiliations:** 1 Institute for Hygiene and Microbiology, University of Würzburg, Würzburg, Germany; 2 Meningococcal Reference Unit, Public Health England, Manchester, United Kingdom; 3 Spanish Reference Laboratory for Meningococci, National Centre for Microbiology, Instituto de Salud Carlos III, Madrid, Spain; 4 National Reference Centre for Bacterial Meningitis, National Medicines Institute, Warsaw, Poland; 5 Dept. of Infectious Diseases, Istituto Superiore di Sanità, Rome, Italy; 6 The Netherlands Reference Laboratory for Bacterial Meningitis, Department of Medical Microbiology, Academic Medical Center, Amsterdam, Netherlands; 7 National Meningitis Reference Laboratory, National School of Public Health, Athens, Greece; 8 National Reference Laboratory for *Neisseria meningitidis*, Department of Laboratory Medicine, Faculty of Medicine and Health, Örebro University, Örebro, Sweden; 9 Department of Infectious Diseases, National Institute of Health Dr. Ricardo Jorge, Lisboa, Portugal; 10 National Reference Laboratory for Meningococcal Infections, National Institute of Public Health, Prague, Czech Republic; 11 Division of Infectious Diseases, Geneva University Hospitals, Geneva, Switzerland; 12 Division for Infection Control and Environmental Health, Norwegian Institute of Public Health, Oslo, Norway; 13 Department of Health Security, National Institute for Health and Welfare (THL), Helsinki, Finland; 14 Department of Epidemiology and Surveillance, National Institute for Public Health and the Environment, Bilthoven, the Netherlands; 15 Division of Communicable Diseases, Federal Office of Public Health (FOPH), Bern, Switzerland

**Keywords:** meningococcal disease, epidemiology, serogroup W, Europe, airborne infections, bacterial infections, Neisseria menigitidis, surveillance

## Abstract

**Background:**

The total incidence of invasive meningococcal disease (IMD) in Europe has been declining in recent years; however, a rising incidence due to serogroup W (MenW), predominantly sequence type 11 (ST-11), clonal complex 11 (cc11), was reported in some European countries.

**Aim:**

The aim of this study was to compile the most recent laboratory surveillance data on MenW IMD from several European countries to assess recent trends in Europe.

**Methods:**

In this observational, retrospective study, IMD surveillance data collected from 2013–17 by national reference laboratories and surveillance units from 13 European countries were analysed using descriptive statistics.

**Results:**

The overall incidence of IMD has been stable during the study period. Incidence of MenW IMD per 100,000 population (2013: 0.03; 2014: 0.05; 2015: 0.08; 2016: 0.11; 2017: 0.11) and the proportion of this serogroup among all invasive cases (2013: 5% (116/2,216); 2014: 9% (161/1,761); 2015: 13% (271/2,074); 2016: 17% (388/2,222); 2017: 19% (393/2,112)) continuously increased. The most affected countries were England, the Netherlands, Switzerland and Sweden. MenW was more frequent in older age groups (≥ 45 years), while the proportion in children (< 15 years) was lower than in other age groups. Of the culture-confirmed MenW IMD cases, 80% (615/767) were caused by hypervirulent cc11.

**Conclusion:**

During the years 2013–17, an increase in MenW IMD, mainly caused by MenW cc11, was observed in the majority of European countries. Given the unpredictable nature of meningococcal spread and the epidemiological potential of cc11, European countries may consider preventive strategies adapted to their contexts.

## Introduction

The total incidence of invasive meningococcal disease (IMD) in Europe has been declining in recent years; however, a rising incidence of IMD caused by serogroup W (MenW) was reported in the United Kingdom (UK) in 2009 [1], then the Netherlands [2] and Sweden [3], following the spread of MenW IMD in South America since 2004 [4]. The isolated strains predominantly belong to the multilocus sequence typing (MLST) defined hypervirulent clonal complex 11 (cc11).

Some patients with MenW:cc11 infection have been reported to present with gastrointestinal symptoms, which is uncommon for patients with IMD caused by meningococci of other serogroups [5]. The case fatality rate (CFR) of MenW IMD was described to be more than twice that of IMD caused by other serogroups, and the proportion of MenW:cc11 in all IMD cases was found to be higher in adults than in infants [6].

In response to the rapid expansion of hypervirulent MenW:cc11 in the UK, a MenACWY conjugate vaccination programme was introduced in August 2015 for all adolescents 13–18 years of age, as well as university students up to 25 years of age [7]. In Italy, the programme was introduced for those 12–18 years of age in 2017 [8] and in the Netherlands for those 13–14 years of age in 2018, with a catch-up vaccination up to the age of 18 [9,10]. MenACWY vaccination has been recommended in Greece since 2011 for those 11–16 years of age [11] and in Austria since 2012 for those 10–13 year of age [12]. In contrast to earlier IMD epidemics where vaccines were not available or became available near the end of the epidemic, such as in New Zealand in the 1990s, several vaccines against MenACWY have been approved and are available in Europe [13,14].

The aim of the present study was to compile the most recent laboratory surveillance data on MenW IMD from several European countries to assess recent trends in Europe.

## Methods

### Setting and data collection

All national reference laboratories active in the European Meningococcal and Haemophilus Disease Society (EMGM) were invited to a workshop on serogroup W and Y meningococci in Europe, taking place in November 2016. This workshop was organised using EMGM’s institutional support from Pfizer, though the company did not have any influence on the outline or content of the meeting.

Reference laboratories from 14 countries were asked to participate in this study. The Czech Republic, England, Finland, Germany, Greece, Italy, the Netherlands, Norway, Poland, Portugal, Spain, Sweden and Switzerland provided the following data in an Excel spreadsheet: the number and age distribution of IMD cases (total and MenW), laboratory surveillance rate of the reported cases and data on cc11 as a surrogate for the W lineages from 2013–16. The participating countries represented over two thirds of the total combined European Union (EU)/European Free Trade Association (EFTA) population.

After identifying and correcting missing or inconsistent numbers, the dataset was compiled and analysed. IMD numbers were based on publicly available national reporting, which is mandatory in all 13 participating countries. On average, 90% of the reported IMD cases were laboratory confirmed by the national reference laboratories, ranging from 60–100% depending on the country. To achieve a high quality standard on the serogrouping data, only MenW cases analysed by reference laboratories were included in the analysis, as serogrouping in peripheral laboratories is not always performed by standardised methods. Reference laboratories reported the number of cases according to the laboratory criteria of the EU case definitions [15]. Typing data were matched at different levels with the statutory notification data. Preliminary 2017 data were collected on IMD and MenW cases, as at the time of writing sequence typing data was not yet available for many countries.

In England, national reporting is based on laboratory-confirmed cases only; therefore, only such cases were included in this study [16]. Dutch and Polish data used in this study were also based on laboratory-confirmed cases. In the Czech Republic, Finland, Germany and Greece the reported numbers were based on mandatory notification data merged with laboratory surveillance data in a national database. As notification data were not available for the number of Spanish IMD cases in 2016–17, this number was extrapolated from the available laboratory surveillance data, representing approximately 80% of the notified cases in the years 2013–15. Population and age distribution data from 12 of the 13 countries were obtained from Eurostat [17]. Data on the English population were acquired from the UK Office for National Statistics [18]. As more recent data were not yet available, the English population number from 2016 was used for 2017.

### Typing

The serogroup was determined by slide agglutination or PCR. Depending on the country, MLST—either on Sanger sequence or next generation sequencing data—was used to determine if strains belonged to cc11. The Bacterial Isolate Genome Sequence Database (BIGSdb) was used for data matching [19].

### Statistics

Average annual percentage change in incidence was calculated for each country using Poisson regression. A p value < 0.05 was considered statistically significant.

## Results

### Incidence of invasive meningococcal diseases cases

From 2013–17, a total of 10,385 IMD cases were reported by the 13 participating countries. Accounting for the slightly growing population (2013: 355.7 million, 2014: 357.4 million, 2015: 358.5 million, 2016: 360.1 million, 2017: 360.8 million), this corresponds to an average annual incidence of 0.58 cases per 100,000 population. Serogroup W was responsible for 1,329 (13%) of the total number of IMD cases (Table 1).

**Table 1:** Number of invasive meningococcal disease cases and number and incidence of laboratory-confirmed MenW cases, by country, 13 EMGM-member countries, 2013–2017 (n = 10,385 cases)

Country	Number of reported IMD cases					MenW cases					MenW incidence					AAPC	p value
	2013	2014	2015	2016	2017	2013	2014	2015	2016	2017	2013	2014	2015	2016	2017		
England^a^	726	628	797	804	704	76	117	201	224	195	0.14	0.22	0.37	0.41	0.35	23%	< 0.001
Germany^b^	345	278	287	339	281	10	10	10	26	26	0.01	0.01	0.01	0.03	0.03	35%	< 0.001
Spain	262	146	210	250^c^	261^c^	6	6	6	21	28	0.01	0.01	0.01	0.05	0.06	62%	< 0.001
Poland^a^	235	180	198	157	204	3	4	7	7	9	< 0.01	0.01	0.02	0.02	0.02	29%	0.056
Italy	172	165	189	228	175	5	8	7	13	9	< 0.01	0.01	0.01	0.02	0.01	17%	0.165
Netherlands^a^	117	77	85	153	198	7	1	9	50	80	0.04	< 0.01	0.05	0.29	0.47	133%	< 0.001
Sweden	74	49	53	62	49	3	2	11	18	17	0.03	0.02	0.11	0.18	0.17	58%	< 0.001
Greece^b^	68	66	57	53	42	2	2	0	1	1	0.02	0.02	0	0.01	0.01	-22%	0.404
Portugal	61	54	66	40	44	1	0	0	1	2	0.01	0	0	0.01	0.02	50%	0.300
Czech Republic**^b^**	59	42	48	43	68	0	2	3	4	3	0	0.02	0.03	0.04	0.03	42%	0.113
Switzerland	50	37	43	51	53	3	6	13	15	18	0.04	0.07	0.16	0.18	0.21	44%	<0.001
Norway	27	18	19	23	17	0	2	0	5	5	0	0.04	0	0.10	0.10	86%	0.016
Finland^b^	20	21	22	19	16	0	1	4	3	0	0	0.02	0.07	0.05	0	13%	0.628
**Total**	**2,216**	**1,761**	**2,074**	**2,222**	**2,112**	**116**	**161**	**271**	**388**	**393**	**0.03**	**0.05**	**0.08**	**0.11**	**0.11**	**35%**	**< 0.001**

AAPC: average annual percentage change; EMGM: European Meningococcal and Haemophilus Disease Society; IMD: invasive meningococcal disease; MenW: serogroup W *Neisseria meningitidis*.

^a^ Based on laboratory-confirmed cases only.

^b^ Reported numbers were based on a database merged from statutory notifications and laboratory surveillance.

^c^ Extrapolated from the available laboratory surveillance data representing ca 80% of the notified cases, as notification data was not yet available.

**Figure 1:**
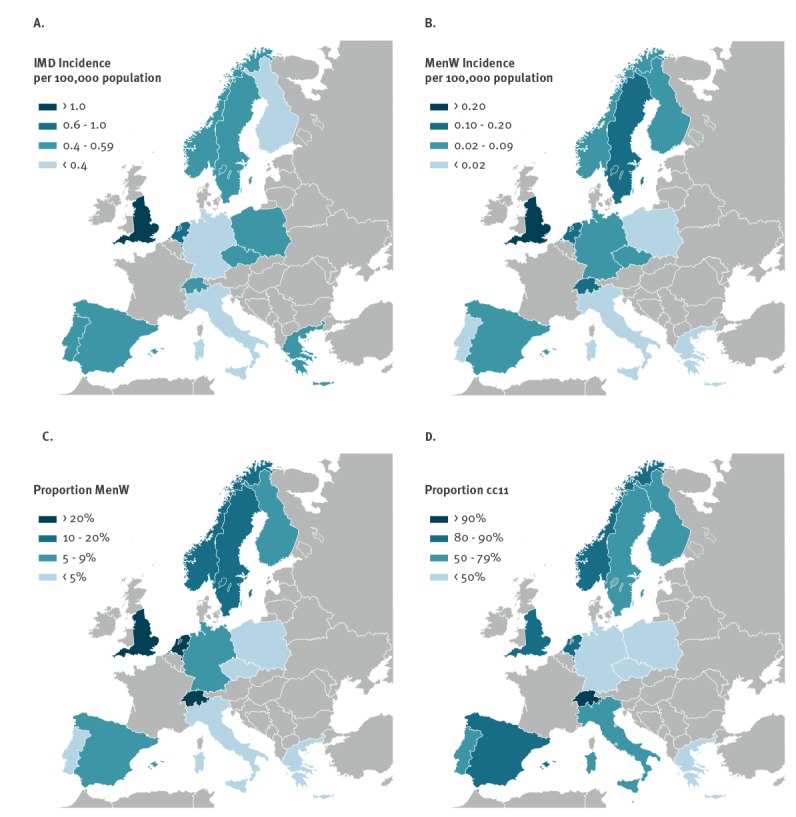
Mean annual incidence of (A) invasive meningococcal disease (IMD) and (B) serogroup W *N. meningitidis* (MenW) IMD per 100,000 population and the proportion of (C) MenW IMD cases and (D) MenW clonal complex 11 IMD cases^a^, by country, 13 EMGM-member countries, 2013–2017

The average annual incidence of overall IMD and MenW IMD varied considerably between individual countries. For IMD, the incidence per 100,000 population ranged from 0.31 cases in Italy to 1.34 in England. For MenW IMD, the incidence per 100,000 population varied from 0.008 cases in Portugal to 0.17 cases in the Netherlands and 0.30 cases in England (Figure 1).

cc11: clonal complex 11; EMGM: European Meningococcal and Haemophilus Disease Society; IMD: invasive meningococcal disease; MenW: serogroup W *Neisseria meningitidis*.

^a^ 2013–16.

Map: Blank map of Europe cropped.svg by Leftcry / CC-BY-2.5.

**Figure 2:**
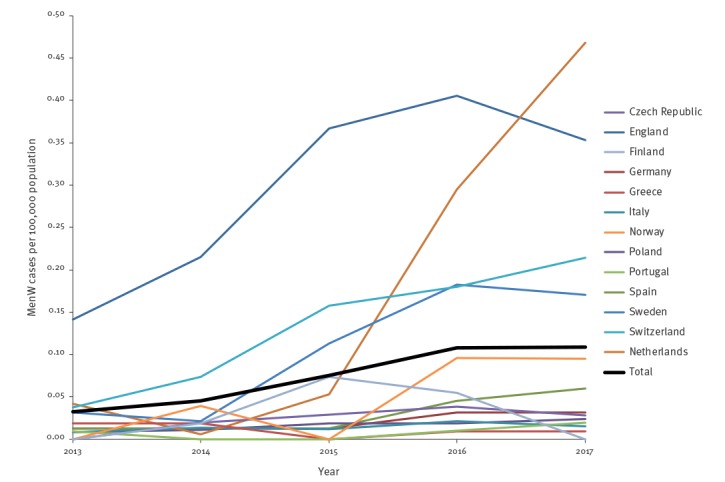
Incidence of invasive meningococcal disease due to serogroup W *Neisseria meningitidis* per 100,000 population, by year and country, 13 EMGM-member countries, 2013–2017 (n = 1,329)

EMGM: European Meningococcal and Haemophilus Disease Society; MenW: serogroup W *Neisseria meningitidis*.

**Figure 3:**
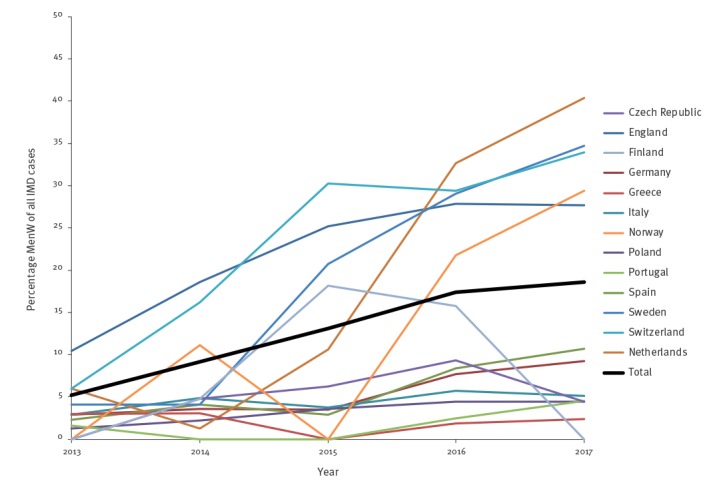
Percentage of serogroup W *Neisseria meningitidis* of all invasive meningococcal disease cases, 13 EMGM-member countries, 2013–2017

EMGM: European Meningococcal and Haemophilus Disease Society; IMD: invasive meningococcal disease; MenW: serogroup W *Neisseria meningitidis*.

While the annual incidence of IMD remained stable in Europe from 2013–17 (2013: 0.62, 2014: 0.49, 2015: 0.58, 2016: 0.62, 2017: 0.59; average annual percentage change (AAPC): 1%; p = 0.214), the incidence of MenW IMD (2013: 0.03, 2014: 0.05, 2015: 0.08, 2016: 0.11, 2017: 0.11; AAPC: 35%; p < 0.001) and the proportion of this serogroup among all IMD (2013: 5%, 2014: 9%, 2015: 13%, 2016: 17%, 2017: 19%; p = 0.002) increased significantly during this time. Significant increases in MenW incidence were observed in the Netherlands (AAPC: 133%; p < 0.001), Norway (AAPC: 86%; p = 0.016), Spain (AAPC: 62%; p < 0.001), Sweden (AAPC: 58%; p < 0.001), Switzerland (AAPC: 44%; p < 0.001), Germany (AAPC: 35%; p < 0.001) and England (AAPC: 23%; p < 0.001). The latter represents 61% of the total number of reported MenW cases in the 13 European countries during the overall study period. Though not statistically significant, there were also trends towards an increasing incidence in some other countries. (Table 1, Figure 2, Figure 3).

The proportion of MenW among all IMD cases varied considerably between countries. The proportion of MenW was lowest in Portugal (2%, 4/265), Greece (2%, 6/286) and Poland (3%, 30/974), while it was highest in Switzerland (24%, 55/234), the Netherlands (23%, 147/630) and England (22%, 813/3,659) (Figure 1).

### Age distribution

**Figure 4:**
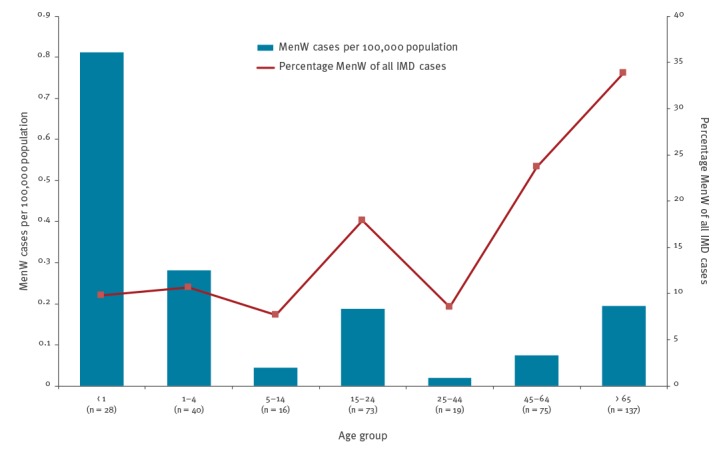
Number and incidence per 100,000 population of invasive meningococcal disease (IMD) caused by serogroup W *Neisseria meningitidis* (MenW), by age group, and percentage of MenW of all IMD cases, 13 EMGM-member countries, 2016

EMGM: European Meningococcal and Haemophilus Disease Society; IMD: invasive meningococcal disease; MenW: serogroup W *Neisseria meningitidis.*

In 2016, MenW IMD cases were overrepresented in the age groups 15–24 years (18% of all IMD cases, 73/408), 45–64 years (24%, 75/316) and ≥ 65 years (34%, 137/405), while the percentage of MenW IMD cases in the vaccination target group of children ≤ 14 years (10%, 84/870), as well as those 25–44 years of age (9%, 19/223), was lower than the average. However, the MenW IMD incidence was still highest in children < 5 years of age (0.8/100,000 population). The lowest proportion of MenW IMD was observed in the age group 5–14 years (8%, 16/209). In absolute numbers, most MenW IMD cases were registered in the age group ≥ 65 years (n = 137). (Figure 4)

### Sequence typing

**Figure 5:**
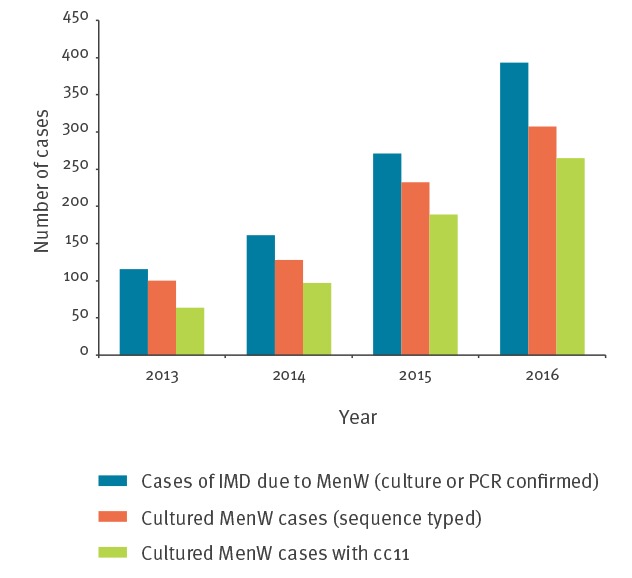
Total serogroup W *Neisseria meningitidis* (MenW) invasive meningococcal disease (IMD) cases, cultured MenW sequence-typed cases and MenW:cc11 cases, 13 EMGM-member countries, 2016

Of the 936 reported cases of MenW IMD between 2013–16, 767 (82%) strain isolates were analysed by MLST and 615 (80%) belonged to cc11. The proportion of cc11 increased from 64% (64/100) in the year 2013 to 86% (265/307) in the year 2016 (Figure 5)

cc11: clonal complex 11; EMGM: European Meningococcal and Haemophilus Disease Society; IMD: invasive meningococcal disease; MenW: serogroup W *Neisseria meningitidis.*

The proportion of sequence-typed MenW strains that belonged to cc11 varied considerably among countries, ranging from one in six in the Czech Republic to 31 in 34 in Switzerland (Figure 1).

## Discussion

Laboratory surveillance and national health authority data from 13 European countries revealed an increase in MenW IMD in the period 2013–17. The MenW IMD incidence differed between participating countries and was highest in England and the Netherlands, as reported previously [1,2]. Significantly increasing incidences and proportions of MenW IMD among all IMD were observed in the majority of the participating countries, while the case numbers in other countries were too small to assess significance.

The increase seen in Europe follows a rising incidence of MenW in South America since 2004 [4]. More recently, a rising MenW incidence has been observed in Australia since 2013 [6] and in Canada since 2015 [20]. An epidemic spread of this MenW strain can be assumed for regions in the Americas, Australia and Europe. Notably, in 2015 an international outbreak related to the World Scout Jamboree in Japan was caused by the MenW:cc11 strain from the UK [21]. This was observed previously for other meningococci serogroups, including serogroup C:cc11 in the 1990s [22], serogroup A since the 1960s [23] and serogroup B since the 1970s [24]. The dynamics of such intercontinental epidemic waves is hardly predictable and the mechanisms behind different dynamics of spread are still unclear. Typing by next generation sequencing revealed the relatedness of the MenW strains from South America, England [25] and the Netherlands [2]. It is possible that cc11 in general has a particular capacity to spread between countries, as these strains have shown this already in the past [21,26-28]. A possible experimental hypothesis is that cc11 strains are maintained in the population by frequent transmission events, despite a short duration of carriage [29].

It is noteworthy that the majority of cases of MenW IMD in Europe between 2013–16 occurred in those ≥ 45 years of age, with the highest number of cases found in patients ≥ 65 years of age. It is unclear whether this is the result of a circulation of strains in those ≥ 45 years of age or if colonised children and teenagers are the major source of transmission to the older population. Indeed, carriage of meningococci is most frequent in adolescents and young adults, with a peak around the age of 20 that decreases with age; however, studies on carriage of meningococci in adults and the elderly are rare [30]. From the existing evidence, it may be suggested that despite infrequent disease, carriage in adolescents and young adults is much more abundant than in infants and toddlers [30]. Whether or not transmission occurs mainly within the affected age strata remains unclear. New carriage studies among all age groups are needed to inform about the MenW spread in Europe.

MenW is vaccine preventable and potent conjugate vaccines have been approved for use in Europe. Many European countries use meningococcal C conjugate vaccines for infant immunisation. A general booster vaccination using a quadrivalent MenACWY vaccine—as introduced in Greece [11], Austria [12], the UK [7], Italy [8] and the Netherlands [9,10]—boosters the waning MenC antibody titres [31] and provides immunity towards MenA, MenW and MenY.

As target groups and coverages vary in different countries (e.g. case numbers in some countries are low) and some programs were introduced quite recently, it is still too early to conclude on the effect of the vaccinations. In theory, by vaccinating adolescents, acquisition of carriage should be prevented and herd protection induced. This may be a cost-effective approach, as it might not be possible to immunise all groups. Public health decision-makers should be aware of the rise of MenW and, taking into account the situations in their respective countries, they may consider including quadrivalent meningococcal ACWY in their adolescent vaccination programmes.

The present study demonstrates the power of laboratory networks to provide surveillance data on short notice. The EMGM has already proved to be a functioning laboratory network on earlier occasions. The present survey relied on Sanger sequencing information, as next generation sequencing has not been rolled out to a sufficient extent in all countries. However, the example of the MenW spread shows that for particular questions this technique is indispensable. The causative agent is a particular derivative of the cc11, which can only be elucidated by high-resolution techniques [3]. The function of BIGSdb as a platform allowing the integration of genome sequence data was first validated using meningococcal sequence data as an example [19]. Future European-level meningococcal disease surveillance databases linking public health reporting data with high-resolution sequencing data would be beneficial for outbreak detection. The respective information technology is available; however, clarification of data protection issues and an unambiguous nomenclature is a prerequisite.

One limitation of this study is the incomplete coverage of Europe. Only 13 of the 32 EU/EFTA countries joined this project. However, more than two thirds of the population of the EU/EFTA area was addressed. It should be noted that in the UK, only England participated. Further, because of varying reporting and surveillance structures, the ways that data were obtained differed between countries, as did the laboratory surveillance coverage. Under-reporting may also have occurred on different levels, as cases of IMD could have been misdiagnosed or misclassified [32]. In addition, cases of MenW IMD may have been missed if they were not serogrouped by a national reference laboratory.

### Conclusion

In conclusion, this study showed a concerning trend of increasing MenW incidence in several European countries, which needs to be followed carefully. Revision of vaccination schedules that takes into account an individual country’s situation may be considered to counteract this trend. Furthermore, this study shows the strength of laboratory networks to combine and evaluate data on short notice. In the case of increasing MenW IMD, serogrouping and basic typing data were indispensable for the evaluation.
